# *De novo* transcriptome sequencing and analysis of genes related to salt stress response in *Glehnia littoralis*

**DOI:** 10.7717/peerj.5681

**Published:** 2018-09-26

**Authors:** Li Li, Mimi Li, Xiwu Qi, Xingli Tang, Yifeng Zhou

**Affiliations:** 1 Institute of Botany, Jiangsu Province and Chinese Academy of Sciences, Nanjing, China; 2 Nanjing Agricultural University, Nanjing, China; 3 Dongtai Institute of Tidal Flat, Nanjing Branch of Chinese Academy of Sciences, Dongtai, China; 4 The Jiangsu Provincial Platform for Conservation and Utilization of Agricultural Germplasm, Nanjing, China

**Keywords:** Gene, *Glehnia littoralis*, Salt stress, Transcriptome analysis

## Abstract

Soil salinity is one of the major environmental stresses affecting plant growth, development, and reproduction. Salt stress also affects the accumulation of some secondary metabolites in plants. *Glehnia littoralis* is an endangered medicinal halophyte that grows in coastal habitats. Peeled and dried *Glehnia littoralis* roots, named Radix Glehniae, have been used traditionally as a Chinese herbal medicine. Although *Glehnia littoralis* has great ecological and commercial value, salt-related mechanisms in *Glehnia littoralis* remain largely unknown. In this study, we analysed the transcriptome of *Glehnia littoralis* in response to salt stress by RNA-sequencing to identify potential salt tolerance gene networks. After *de novo* assembly, we obtained 105,875 unigenes, of which 75,559 were annotated in public databases. We identified 10,335 differentially expressed genes (DEGs; false discovery rate <0.05 and |log_2_ fold-change| ≥ 1) between NaCl treatment (GL2) and control (GL1), with 5,018 upregulated and 5,317 downregulated DEGs. To further this investigation, we performed Gene Ontology (GO) analysis and the Kyoto Encyclopaedia of Genes and Genomes (KEGG) pathway analysis. DEGs involved in secondary metabolite biosynthetic pathways, plant signal transduction pathways, and transcription factors in response to salt stress were analysed. In addition, we tested the gene expression of 15 unigenes by quantitative real-time PCR (qRT-PCR) to confirm the RNA-sequencing results. Our findings represent a large-scale assessment of the *Glehnia littoralis* gene resource, and provide useful information for exploring its molecular mechanisms of salt tolerance. Moreover, genes enriched in metabolic pathways could be used to investigate potential biosynthetic pathways of active compounds by *Glehnia littoralis*.

## Introduction

High soil salinity has caused extremely negative effects on global agricultural production and ecological environments. Plant growth, development, metabolism, and protein functions can be retarded and inhibited under salt stress (Carillo et al., 2011). High salinity affects plants during two phases, the early and rapid osmotic stress phase and the later and slower ionic toxicity phase (Munns & Tester, 2008). To resist damage caused by salt stress and survive in high-salt conditions, plants have gradually formed a series of complex mechanisms including osmotic regulation, Na^+^ exclusion, and Na^+^ compartmentalisation (Deinlein et al., 2014; Munns & Tester, 2008). A variety of signal transduction pathways are involved in plant responses to salt stress, including the phospholipid signaling pathway (Hong et al., 2016), salt overly sensitive (SOS) pathway (Qiu et al., 2002; Quan et al., 2007), abscisic acid (ABA) pathway (Hauser et al., 2017), calcium-dependent protein kinase (CDPK) pathway, and mitogen-activated protein kinase (MAPK) cascade pathway (Zhu, 2016). These pathways interact to form signal transduction networks in plant responses to salt stress. Numerous breakthroughs have been reported in the study of plant salt tolerance; however, many salt-related functional genes remain to be discovered and identified.

*Glehnia littoralis* Fr. Schmidt ex Miq. belongs to the Umbelliferae family. *Glehnia littoralis* germplasm resources are distributed in Japan, Russia, China, the Korean Peninsula, the Pacific Rim, and coastal areas of the United States. It is an endangered halophyte shown to exhibit salt tolerance (Peng et al., 2014), and has traditional and medicinal uses. Peeled and dried *G. littoralis* roots, named Radix Glehniae, have been used as a traditional Chinese herbal medicine for moistening lungs, removing phlegm, curing respiratory and gastrointestinal disorders, and anti-inflammation. This herbal medicine is also a major tonic component in anti-aging and health promotion prescriptions. Coumarins and polyacetylenes are the primary active constituents of *G. littoralis* (Yuan et al., 2002). Although *G. littoralis* has great ecological and commercial value, salt-related mechanisms in *G. littoralis* remain largely unknown. Thus far, most studies of salt tolerance in *G. littoralis* have focused on anatomical and morphological adaptations to high-salinity environments (Voronkova et al., 2011); for example, *Glehnia littoralis* leaves exhibit dorsoventral structure and have developed secretory trichomes that remove excess salts. However, the salt tolerance mechanisms and functional gene information of *G. littoralis* have not been explored.

In recent years, *de novo* transcriptome assembly and annotation of non-model plant species, especially those with unknown genomic sequences, have become possible through RNA sequencing (RNA-Seq) technology (Ward, Ponnala & Weber, 2012; Xiao et al., 2013). Transcriptome analysis has greatly facilitated the discovery of putative functional genes or proteins involved in various plant biological processes (Cao & Deng, 2017; Lu et al., 2017; Wang et al., 2017). Comprehensive transcriptome analysis has been performed on various plant species in response to various abiotic stresses, such as cold, drought, salt, and hormone stresses, and has provided insight into their functional gene expression and regulation, signal transduction networks, and metabolite biosynthetic pathways (Postnikova, Shao & Nemchinov, 2013; Wang et al., 2013; Wang et al., 2017; Zhang et al., 2014a; Zhou et al., 2017). For example, recently in *Litchi chinensis*, 73,117 unigenes were assembled and 11,741 unigenes were identified as being both chilling and ROS responsive genes (CRRGs) (Lu et al., 2017). Some of these CRRGs are involved in flowering, plant hormone signal transduction and plant hormone biosynthesis, and exhibit relationships within genes co-expression networks. Zhang et al. (2014a) performed transcriptome analysis of *Populus pruinosa* in response to salt stress by constructing six libraries from control and salt-treatment calli at different time-points. They identified 9,216 differentially expressed genes (DEGs) and found that during the salt treatment, most of the DEGs were activated early (within 24 h) and stabilized after 48 h.

Halophytes are ideal plants for studying plant salt tolerance genetics using transcriptome sequencing. Comparative transcriptome analyses have previously been reported for a few halophytes, such as *Halogeton glomeratus* (Yao et al., 2018; Wang et al., 2015), *Iris lactea var. chinensis* (Gu et al., 2018), *Kochia sieversiana* (Zhao et al., 2017), *Nitraria sibirica* Pall. (Li et al., 2017a), *Suaeda maritima* (Gharat et al., 2016), *Sporobolus virginicus* (Yamamoto et al., 2015), and *Mesembryanthemum crystallinum* (Oh et al., 2015). In one such study, Wang et al. (2015) compared the transcriptomes of *H. glomeratus* exposed to NaCl for 6, 12, 24, and 72 h and discovered 2,223, 5,643, 7,510 and 10,908 DEGs, respectively. Later, Yao et al. (2018) analysed root transcriptomes of *H. glomeratus* under salt stress and identified the core DEGs regulating Na^+^ uptake and transport in the roots. *G. littoralis* is a typical halophyte belonging to the family Umbelliferae. To date, no genomic or transcriptome sequencing information about salt stress responses has been reported for any halophytes belonging to Umbelliferara, including *G. littoralis*. As a medicinal halophyte, salt-associated molecular mechanisms in *G. littoralis* are important to a comprehensive understanding of this plant. In the current study, we performed comparative transcriptome sequencing of *G. littoralis* under NaCl treatment, and identified a total of 105,875 unigenes, 71.37% of which were annotated in public databases. These transcriptome analysis results will help to promote genomics and genetics studies on salt-associated mechanisms and on secondary metabolite biosynthesis in *G. littoralis*.

## Materials & Methods

### Plant growth and treatment

*G. littoralis* plant material was collected from Tannanwan Beach, Pingtan, Fujian Province, China (25°26′1.86″N, 119°45′14.4″E) and pot-cultured at the Institute of Botany, Jiangsu Province and Chinese Academy of Sciences, Nanjing, China. *Glehnia littoralis* seedlings were grown in nutrient-enriched sandy soil under a 14-h light (26 °C)/10-h dark (22 °C) photoperiod. After 3 months of pot growth, the seedlings were treated with 200 mM NaCl for 24 h. Seedlings without NaCl treatment (0 h) were used as a control. The shoots and roots were harvested together and immediately frozen in liquid nitrogen and stored at –80 °C for subsequent RNA extraction. The control and salt-treated samples were labelled GL1 and GL2, respectively. Six biological replicates were harvested and mixed together for the RNA extraction of each sample.

### RNA extraction and cDNA library preparation

Total RNA of *G. littoralis* was extracted from each sample using Trizol Reagent (TakaRa, Dalian, China) following the manufacturer’s instructions. Genomic DNA was removed using DNase I (TaKaRa, Dalian, China). The quality of RNA was determined using an Agilent 2100 Bioanalyzer (Agilent Technologies, Inc., Santa Clara, CA, USA) and the quantity was determined using a NanoDrop ND-2000 (Thermo Scientific, Wilmington, MA, USA). Only high-quality RNA samples (OD260/280 = 1.8∼2.2, OD260/230 ≥ 2.0, RIN ≥ 6.5, 28S:18S ≥ 1.0, >10µg) were used to construct the sequencing libraries. RNA purification, reverse transcription, library construction and sequencing were performed at Shanghai Majorbio Bio-pharm Biotechnology Co., Ltd. (Shanghai, China) according to the manufacturer’s instructions (Illumina, San Diego, CA, USA) as follows. The *G. littoralis* RNA-seq transcriptome libraries were prepared using the Illumina TruSeq™ RNA sample preparation Kit. Poly(A) mRNA was purified from total RNA using oligo-dT-attached magnetic beads and then randomly fragmented into short fragments (about 200 bp) by metal ions. Taking these short fragments as templates, double-stranded cDNA was synthesized with a SuperScript double-stranded cDNA synthesis kit (Invitrogen, Carlsbad, CA, USA) with random primers. Then, the double-stranded cDNA was further end-repaired and A-tailed, and indexed adapters were ligated. After PCR amplification for 15 PCR cycles, cDNA libraries were selected for cDNA target fragments of 200–300 bp on 2% Low Range Ultra Agarose. After quantification with a TBS380 Mini-Fluorometer, two RNA-seq libraries were sequenced in single lane on an Illumina Hiseq 2000 sequencer (Illumina, San Diego, CA, USA) for 2 × 100 bp paired-end reads.

### *De novo* transcriptome assembly and unigene annotation

After sequencing, the raw paired-end reads were trimmed and quality-controlled (QC) by SeqPrep (https://github.com/jstjohn/SeqPrep) and Sickle (https://github.com/najoshi/sickle) using default parameters. The reads were filtered as follows: the adapter was removed from the reads; low-quality bases (quality value < 20) at the 3′ end of the sequence were cut; if the quality value of residual sequence was still less than 10, the entire sequence was removed, otherwise the read was retained; reads that contained too many Ns (≥10%) were removed; and reads that were less than 20 bp in length after adapter discarding and quality control were removed. Clean data from the two samples (GL1 and GL2) were then used for *de novo* assembly with Trinity (http://trinityrnaseq.sourceforge.net/) (Grabherr et al., 2011).

To annotate unigenes, all of the assembled transcripts were searched against public databases including the non-redundant protein database (Nr), euKaryotic Orthologous Groups (KOG), and the Kyoto Encyclopaedia of Genes and Genomes (KEGG), using an *E*-value ≤1 × 10^−5^ to obtain optimal functional annotation. The BLAST2GO (http://www.blast2go.com/b2ghome) (Conesa et al., 2005) program was used to obtain the Gene Ontology (GO) annotations of unique assembled transcripts for describing biological process, molecular function and cellular component. Metabolic pathway analysis was performed using KEGG (http://www.genome.jp/kegg/).

The original data were deposited into the National Center for Biotechnology Information (NCBI) Sequence Read Archive (SRA) (accession number: SRX547159). This Transcriptome Shotgun Assembly project has been deposited at DDBJ/EMBL/GenBank under the accession GGSB00000000. The version described in this paper is the first version, GGSB01000000.

### Analysis of differentially expressed genes (DEGs)

The RSEM (RNA-Seq by Expectation-Maximization, http://deweylab.biostat.wisc.edu/rsem/) method was used to quantify gene abundances (Li & Dewey, 2011). The R statistical package software edgeR (Empirical Analysis of Digital Gene Expression in R, http://www.bioconductor.org/packages/2.12/bioc/html/edgeR.html) (Robinson, Mccarthy & Smyth, 2010) was used for differential expression analysis and identification of DEGs. Read counts were input to the edgeR software and the screening criteria for significant DEGs was set to a false discovery rate (FDR) <0.05 and —log_2_ fold-change— ≥ 1. Gene expression levels were reported in fragments per kilobase of exon per million mapped reads (FPKM) (Mortazavi et al., 2008). The log_2_FPKM values of the DEGs (using 0.001 instead of 0) were used to generate heat maps with HemI software (Deng et al., 2014). In addition, GO and KEGG enrichment analyses were performed to identify which DEGs were significantly enriched in GO terms and metabolic pathways compared with the whole-transcriptome background with a Bonferroni-corrected *P*-value ≤ 0.05 GO functional enrichment and KEGG pathway analyses of DEGs were carried out by Goatools (https://github.com/tanghaibao/Goatools) and KOBAS (http://kobas.cbi.pku.edu.cn/) (Xie et al., 2011). Differentially expressed transcription factors were then identified by alignment to the Plant Transcription Factor Database PlnTFDB (http://plntfdb.bio.uni-potsdam.de/v3.0/) and PlantTFDB (http://planttfdb.cbi.pku.edu.cn).

### qRT-PCR analysis of DEGs

*Glehnia littoralis* total RNA was extracted according to the method described above. Some up- or down-regulated DEGs, which are involved in salt response and secondary metabolite biosynthesis, were selected for quantitative real-time PCR (qRT-PCR) assays. qRT-PCR was performed according to a protocol described previously (Wang et al., 2017) on a qTOWER 2.2 Real-Time PCR System (Analytik Jena AG, Jena, Germany) using SYBR Premix Ex Taq™ II (Tli RNaseH Plus) (Takara, Dalian, China). Expression of the *Actin* gene of *Glehnia littoralis* was used as the internal reference. Relative gene expression was analysed using the 2^−^^△△^^CT^ method (Livak & Schmittgen, 2001). All qRT-PCR primers are listed in Table S1.

## Results

### *G. littoralis* transcriptome sequencing profile under NaCl treatment

**Figure 1:**
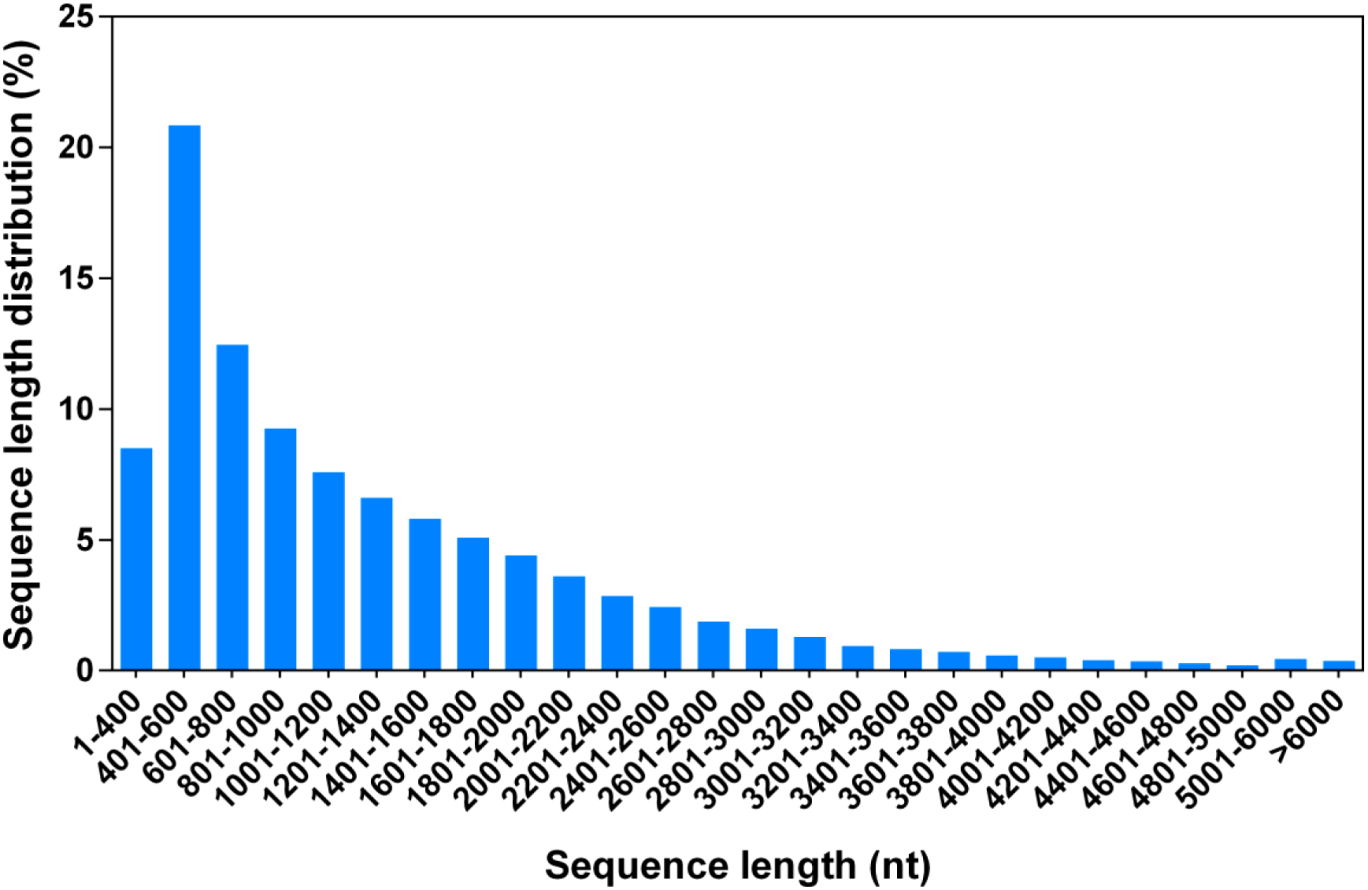
Length distribution of all *G. littoralis* unigenes.

To perform a comprehensive analysis of the *G. littoralis* transcriptome under salt treatment, we constructed two RNA-Seq libraries, representing non-salt treatment (0 h, GL1) and 24 h of 200 mM NaCl treatment (GL2), using the Illumina HiSeq 2000 platform. The GL1 and GL2 libraries produced 5.31 and 5.09 Gb of clean data from the paired-end reads with Q20 percentages of 96.7% and 96.5%, respectively (Table 1). The clean reads from the GL1 and GL2 libraries were used for *de novo* assembly with the Trinity package. The Trinity method determines splice isoforms, distinguishes transcripts from recent duplicates, and identifies allelic variants (Grabherr et al., 2011); thus, each isoform corresponded to a unigene in the present dataset. In total, there were 105,875 unigenes (isoforms) of between 351 and 15,667 bp in sequence assembly, with a mean length of 1,314.44 bp (Fig. 1, Table 2).

**Table 1:** Summary of *G. littoralis* RNA sequencing (RNA-Seq).

**Samples**	**Raw reads**	**Clean reads**	**Clean bases**	**Q20 percentage (%)**
GL1 (control)	54568070	53715849	5.31 Gbyte	96.70%
GL2 (NaCl)	52469596	51566060	5.09 Gbyte	96.50%

**Table 2:** Summary of *G. littoralis* transcriptome assembly.

**Type**	**Number**
Total genes	53,092
Total unigenes	105,875
Total residues	1.39E+08
Average length	1,314.44
Largest unigene	1,5667
Smallest unigene	351

### Sequence annotation and classification

**Figure 2:**
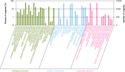
Gene ontology (GO) classification of the unigenes. Based on functional annotation, these unigenes were grouped into three categories: biological process, cellular component, and molecular function.

To annotate the total unigenes, sequences were aligned using public databases (Table 3). In total, 75,535 unigenes (71.34%) were matched in the NR database, 25,871 unigenes (24.44%) in the KOG database, 47,043 unigenes (44.43%) in the GO database, and 24,864 (23.48%) in the KEGG database (Table 3). Taken together, 75,559 unigenes (71.37%) were successfully annotated in at least one of the databases (Table 3). In the GO annotation analysis, 47,043 unigenes were found and divided into three groups. The proportions of unigenes aligned in each GO term are shown in Fig. 2. Based on sequence homology with genes with known functions, these unigenes could be assigned to one or more ontologies, including 132,455 unigenes assigned to the biological process category, 100,205 unigenes assigned to the cellular component category, and 58,269 unigenes assigned to the molecular function category (Table S2). Some unigenes were assigned to more than one GO term, that is, a unigene may belong to several GO term notes and each GO term may correspond to multiple genes. Cellular process (28,273) and metabolic process (27,405) were highly represented groups within the biological process category. In the cellular component category, cell (23,485), cell part (23,483), and organelle (17,311) were highly represented groups. Moreover, catalytic activity (26,412) and binding (24,277) constituted the largest proportion in the molecular function category (Fig. 2, Table S2).

**Table 3:** Functional annotations of all unigenes found in the public databases.

**Annotation**	**Number of unigenes**	**Percentage (%)**
Annotated in NR	75,535	71.34356553
Annotated in KOG	25,871	24.43541913
Annotated in GO	47,043	44.4325856
Annotated in KEGG	24,864	23.48429752
Annotated in at least one database	75,559	71.36623377
Total unigenes	105,875	100

**Figure 3:**
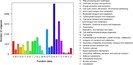
euKaryotic Orthologous Groups (KOG) classification of unigenes. Functional predictions of unigenes were classified into at least 25 function classes according to the KOG database.

**Figure 4:**
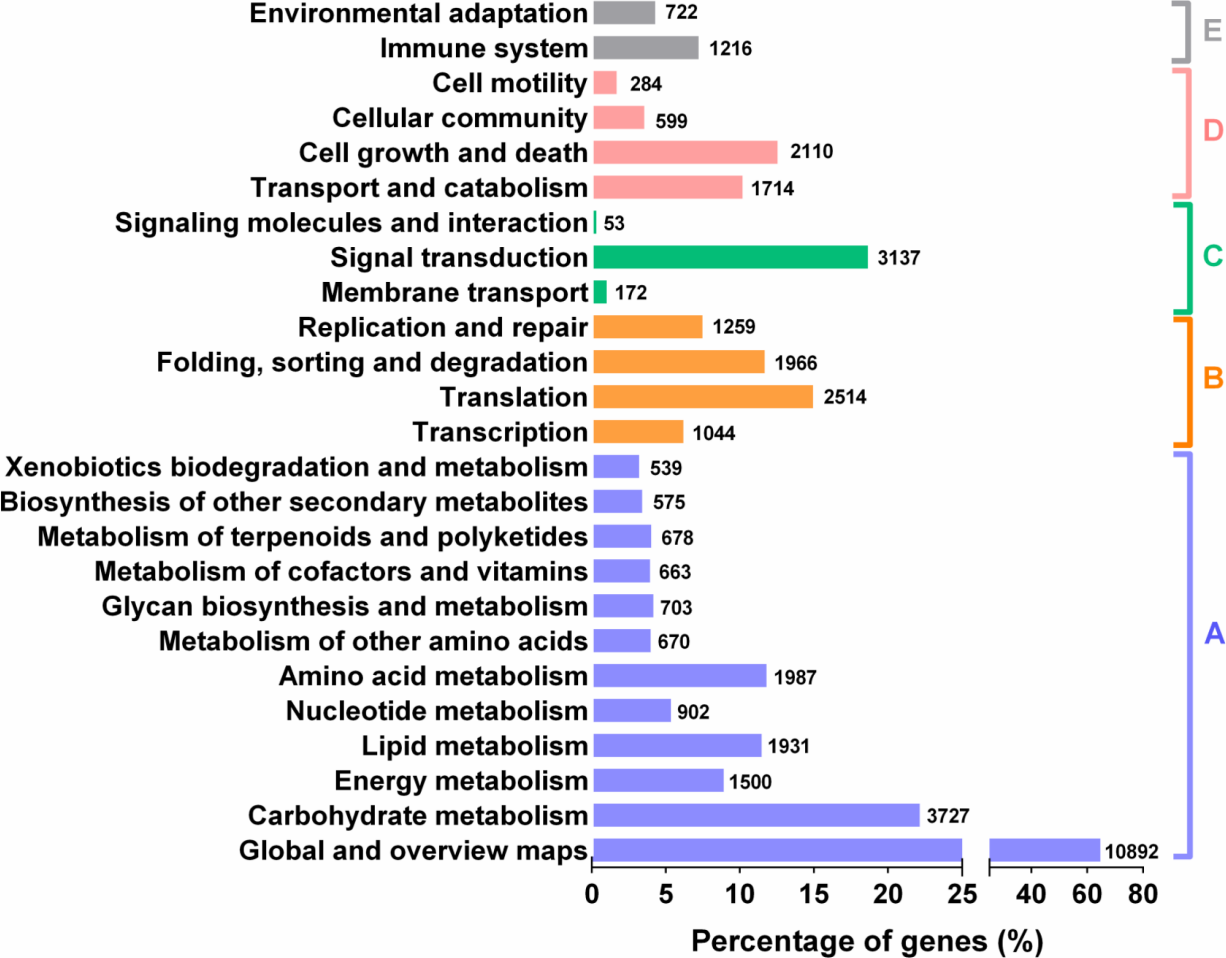
Kyoto Encyclopaedia of Genes and Genomes (KEGG) metabolism pathway classification of unigenes. Data were classified into in five main categories: (A) metabolism, (B) genetic information processing, (C) environmental information processing, (D) cellular processes, and (E) organismal systems. Bars represent the number of *G. littoralis* unigene matches in each category.

In addition, 25,871 unigenes had significant matches in the KOG database for functional prediction and classification (Fig. 3, Table 2). Among the 25 KOG categories, the cluster for general function prediction only (5,358) was the largest category, followed by signal transduction mechanisms (3,494), posttranslational modification, protein turnover, chaperones (2,630), and transcription (1,766). Cell motility (five) and extracellular structures (70) were the smallest categories (Fig. 3, Table S3). We also performed KEGG pathway analysis of the unigenes. In total, 41,556 unigenes participated in 25 predicted metabolic pathways (Fig. 4; Table S4), which were divided into five categories, including metabolism, genetic information processing, environmental information processing, cellular processes, and organismal systems. The largest category was metabolism, including global and overview maps (10,892), followed by carbohydrate metabolism (3,727), amino acid metabolism, (1,987), lipid metabolism (1,931), energy metabolism (1,500), nucleotide metabolism (902), glycan biosynthesis and metabolism (703), metabolism of terpenoids and polyketides (678), metabolism of other amino acids (670), metabolism of cofactors and vitamins (663), biosynthesis of other secondary metabolites (575), and xenobiotics biodegradation and metabolism (539) (Fig. 4, Table S4).

### Identification of DEGs under NaCl treatment

**Figure 5:**
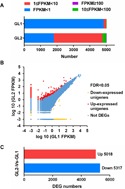
Expression analysis of differentially expressed genes (DEGs) in *Glehnia littoralis* under NaCl treatment. (A) Changes in fragments per kilobase of exon per million mapped reads (FPKM) value of unigenes in the control (GL1) and NaCl (GL2) samples. (B) Scatter plots of gene expression. (C) Number of DEGs up- or downregulated in the samples.

The clean reads from GL1 (control) and GL2 (NaCl) were mapped to the assembled transcriptome sequence to acquire read counts data using RSEM. Then, DEGs between two samples were identified using edgeR package with FDR <0.05 and —log_2_ fold-change— ≥ 1. Unigene expression was calculated using the mappable reads, and the results were normalised to FPKM values. For each sample, more than 99% of FPKM values were between 1 and 100 (Fig. 5A). In total, we identified 10,335 DEGs between the GL1 and GL2 libraries; 5,018 DEGs were upregulated and 5,317 DEGs were downregulated (Figs. 5B, 5C). Next, all DEGs were subjected to functional-enrichment GO analysis: 5,055 DEGs were significantly enriched in GO terms (Table S5) and were classified into three main categories, namely biological process, cellular component, and molecular function. In the biological process category, cellular process (2,715) made up the majority, followed by metabolic process (2,659) and single-organism metabolic process (2,421). In the cellular component category, organelle part (657) and intracellular organelle part (651) were predominant. In the molecular function category, catalytic activity (2,812) and binding (2,641) were prominently represented (Table S6).

**Figure 6:**
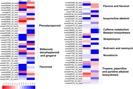
Heat map showing the expression profiles of DEGs in secondary metabolism pathways according to KEGG enrichment. Colour bar presents log2 ratios of FPKM values.

We also performed an analysis of KEGG pathway enrichment in DEGs. Of the 10,335 DEGs, 1,188 unigenes were assigned and divided into 178 KEGG pathways (Tables S5,  S7). Ribosome (123), lysosome (73), spliceosome (67), endocytosis (53) and plant-pathogen interaction (46) were highly represented pathways under salt stress (Table S7). Additionally, we identified the DEGs involved in the pathways of secondary metabolites biosynthesis and generated a heat map of these DEGs (Fig. 6). Phenylpropanoid biosynthesis (20) represented the largest proportion, followed by tropane piperidine and pyridine alkaloid biosynthesis (11), flavonoid biosynthesis (10), and isoquinoline alkaloid biosynthesis (eight). The annotation details of these DEGs are included in Table S8.

### Genes involved in salt response

**Figure 7:**
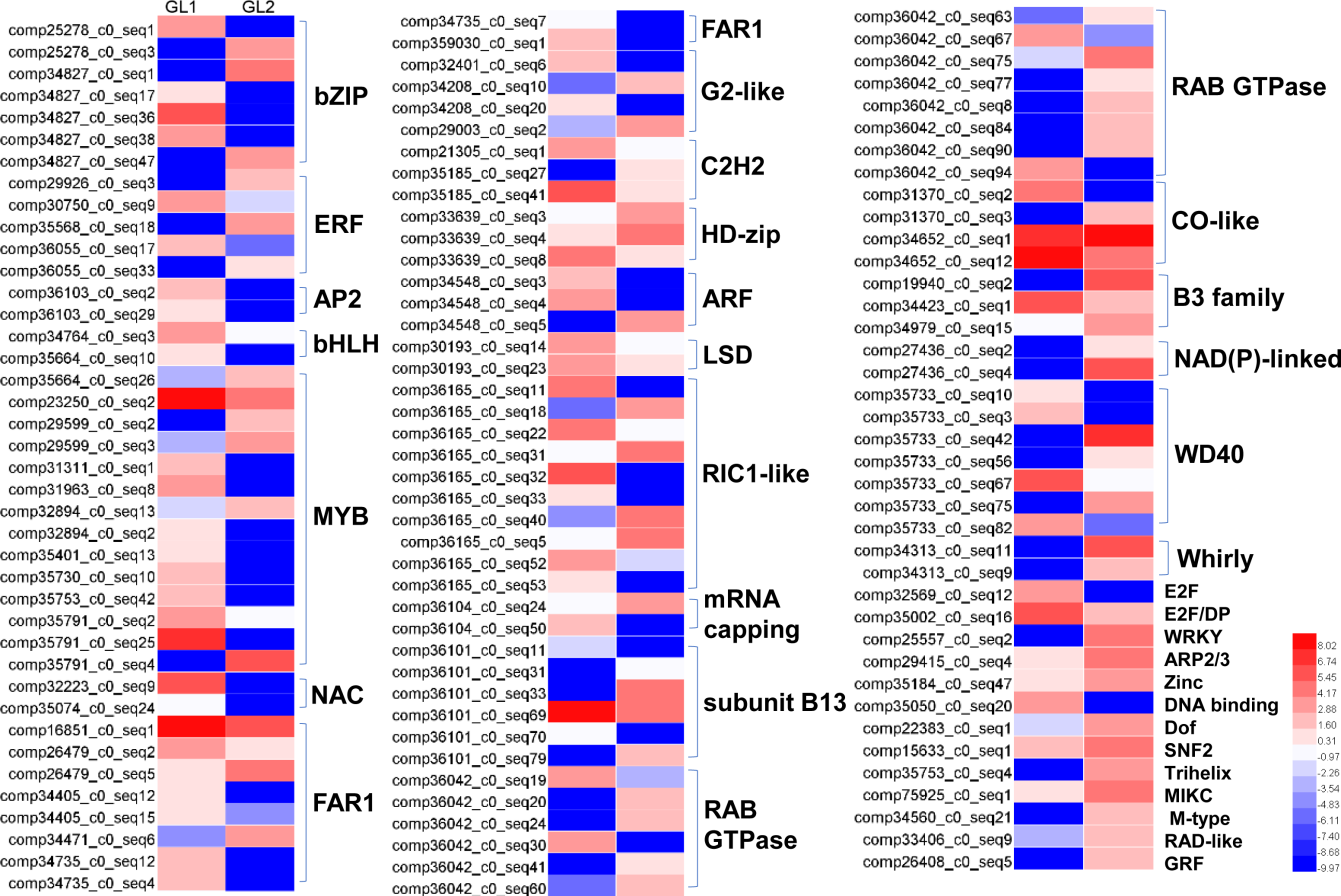
Heat map showing the expression profiles of DEG transcription factors. The colour bar presents log2 ratios of FPKM values.

**Figure 8:**
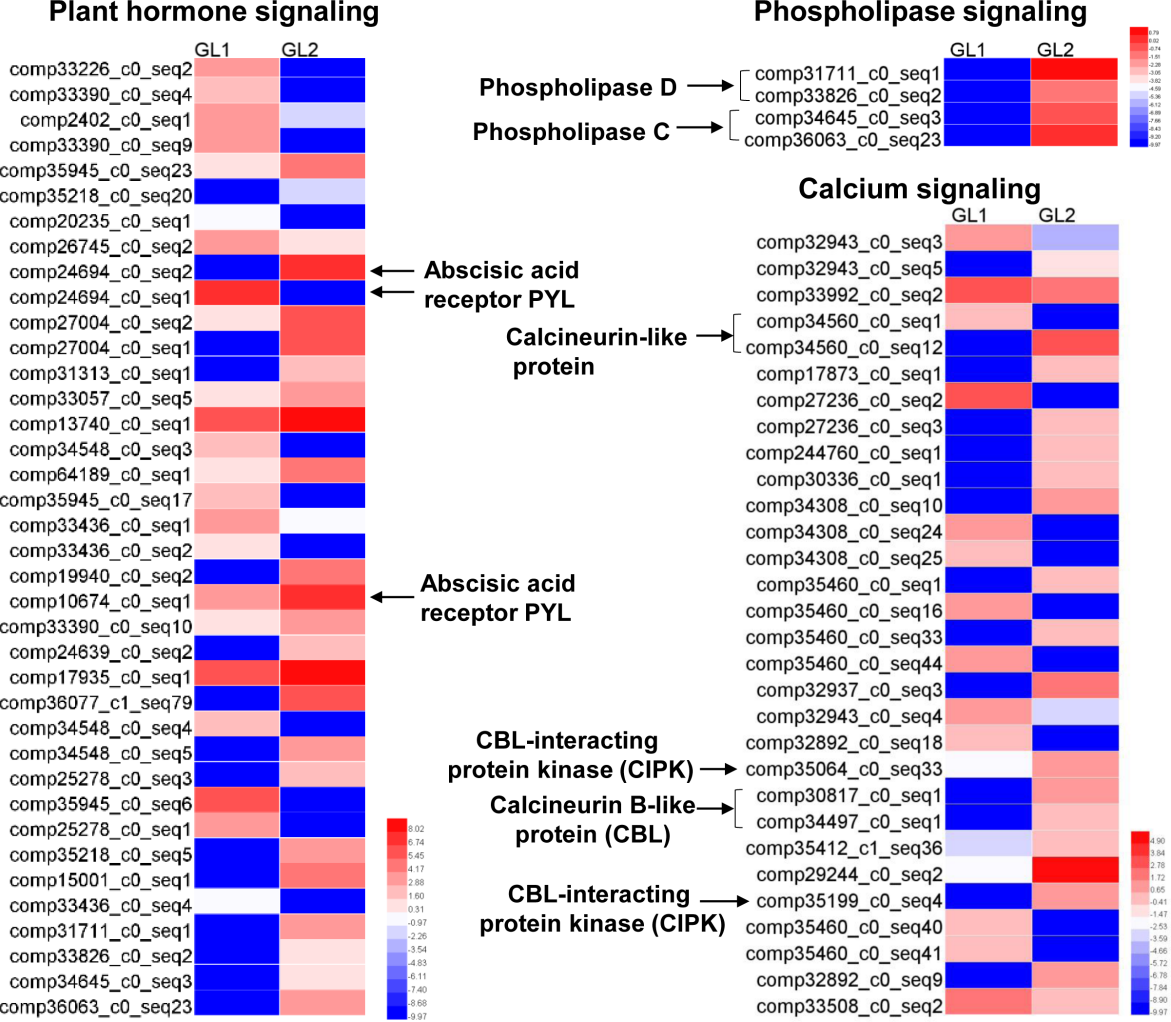
Heat map showing the expression profiles of DEGs in some salt-response signaling pathways. The colour bar represents log2 ratios of FPKM values.

To identify genes responsible for salt response in *Glehnia littoralis*, we performed a BLAST search against the public databases. We found 1,661 unigenes encoding transcription factors (TF); 151 of these were DEGs (Table S5). These DEGs encoding transcription factors were divided into 36 types including MYB, basic leucine zipper (bZIP), ethylene responsive factor (ERF), helix-loop-helix (bHLH), and NAC. Among these, 71 DEGs were upregulated in response to NaCl treatment (Fig. 7). Additionally, unigenes of plant signal transduction pathways were collected from the DEGs according to the KEGG pathway. For example, 38 DEGs related to plant hormone signaling, 30 DEGs related to calcium signaling, and four DEGs related to phospholipase signaling were found to be responsive to NaCl treatment of *Glehnia littoralis* (Table S9). The heat map of these unigenes is displayed in Fig. 8.

### Confirmation of DEGs by qRT-PCR

**Figure 9:**
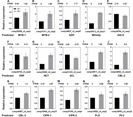
Expression patterns of 15 selected unigenes between *Glehnia littoralis* control and NaCl samples. Expression patterns of 15 selected unigenes between Glehnia littoralis control and NaCl samples. (A) Unigene comp29599_c0_seq3 (MYB-1). (B) Unigene comp31311_c0_seq2 (MYB-2). (C) Unigene comp34827_c0_seq47 (bZIP). (D) Unigene comp34313_c0_seq11 (Whirkly). (E) Unigene comp34764_c0_seq3 (bHLH). (F) Unigene comp30750_c0_seq9 (ERF). (G) Unigene comp35259_c0_seq3 (HCT). (H) Unigene comp51216_c0_seq1 (4CL). (I) Unigene comp30817_c0_seq1 (CBL-1). (J) Unigene comp34560_c0_seq12 (CBL-2). (K) Unigene comp34497_c0_seq1 (CBL-3). (L) Unigene comp35064_c0_seq33 (CIPK-1). (M) Unigene comp35199_c0_seq4 (CIPK-2). (N) Unigene comp33826_c0_seq2 (PLD). (O) Unigene comp36063_c0_seq23 (PLC). Unigene expression was analysed by qRT-PCR using Actin gene as an internal reference. Data represent means ± standard deviation (SD) from three biological replicates (three technical replicates per biological replicate). Repetition of the experiment produced similar results. The FPKM value obtained by RNA sequencing is indicated above each graph (A–O).

To confirm the accuracy and reliability of the transcriptome analysis data, 15 candidate unigenes associated with salt response and secondary metabolites were randomly selected for qRT-PCR assays (Fig. 9). These unigenes included predicted transcription factors and functional proteins. For example, MYB, bZIP, Whirkly, bHLH, and ERF transcription factors, calcineurin B-like protein (CBL), CBL-interacting protein kinase (CIPK), and phospholipases D and C were tested. Additionally, genes involved in second metabolites such as hydroxycinnamoyl transferase (HCT) and 4-coumarate-CoA ligase-like (4CL) were tested. As shown in Fig. 9, trends in the expression of these unigenes determined by qRT-PCR were consistent with those shown through RNA-Seq data, although the fold-changes detected by RNA-Seq and qRT-PCR did not match perfectly. Overall, these results suggest that our *Glehnia littoralis* transcriptome analysis using RNA-Seq was reliable.

## Discussion

The medicinal plant *Glehnia littoralis* exhibits salt tolerance consistent with its original habitat. At present, little genomic information is available for *Glehnia littoralis*. In this study, we performed *de novo* transcriptome analysis of *Glehnia littoralis* using the Illumina HiSeq 2000 platform to investigate the salt tolerance mechanism and functional genes of *Glehnia littoralis*. Although only two libraries were constructed for *de novo* assembly and analysis with and without NaCl treatment, several plants were pooled for each sequencing treatment to reduce background difference among the samples as much as possible. Certainly, more sequencing replicates could be conducive to statistical screening and some outlier data could be removed by calculating the correlation among samples. Whatever, the expression results of the sequencing should be considered for validation by qRT-PCR and further gene function studies. In this study, our qRT-PCR results also supported the sequencing data. In total, we identified 10,335 DEGs and these DEGs were functionally categorised into a variety of physiological and molecular processes, which revealed the conserved mechanisms of salt responsive genes in *Glehnia littoralis*.

Plants subjected to salt stress display complex molecular responses such as stress gene expression, transcriptional regulation, and signal transduction networks (Deinlein et al., 2014). Therefore, we also focused on DEGs enriched in KEGG pathways under salt stress, and provided detailed information for genes in *Glehnia littoralis* associated with plant signal transduction pathways such as hormone signaling, calcium signaling, and phospholipase signalling (Fig. 8). ABA is an essential phytohormone that regulates plant stress response to environmental stimuli (Peleg & Blumwald, 2011). Drought and salinity cause osmotic stress in plants, and ABA is synthesised rapidly to trigger ABA-inducible gene expression, stomatal closure, and transpiration reduction to defend against water deficiency (Wilkinson & Davies, 2010; Yamaguchishinozaki & Shinozaki, 2006). In this study, we identified 524 unigenes involved in plant hormone signal transduction pathways. Of them, 34 DEGs including three pyrabactin resistance 1-like (PYL) ABA receptor genes were identified between the control and NaCl treatment samples (Fig. 8, Tables S7, S9). A previous study had shown that overexpression of PYL could enhance drought resistance and drought-induced leaf senescence in both *Arabidopsis* and rice by limiting transpirational water loss and water condition, thus generating an osmotic potential gradient leading water to preferentially flow to developing tissues (Zhao et al., 2016).

In addition, 30 DEGs were assigned to the calcium signaling pathway in *Glehnia littoralis* (Fig. 8, Tables S7, S9). Ca^2+^ signals are vital transducers and regulators in plant responses to environmental stimuli (Kudla, Batistic & Hashimoto, 2010). Typically, the salt-tolerance SOS3-SOS2-SOS1 pathway demonstrates the significance of Ca^2+^ signaling for excluding excess Na^+^ and maintaining cellular ion homeostasis (Qiu et al., 2002). SOS3 is a calcium sensor, belonging to the category of calcineurin B-like proteins (CBLs). SOS3 interacts with SOS2, a CBL-interacting protein kinase (CIPK), and the SOS3-SOS2 complex activates SOS1 (Na^+^/H^+^ antiporter) to promote the exclusion of Na^+^ to extracellular regions (Qiu et al., 2002). In Arabidopsis, AtCBL4 and CIPK24 correspond to SOS3 and SOS2, respectively (Gong et al., 2004). Furthermore, it has been demonstrated that AtSCABP8 (SOS3-like calcium binding protein8/CBL10) interacts with SOS2 and regulates SOS1 in the shoot response to salt stress (Quan et al., 2007). The plant CBL and CIPK gene families encompass several members; different CBL-CIPK complexes regulate different target proteins to respond to environmental stimuli and developmental needs (Kanwar et al., 2014; Liu et al., 2013; Pandey et al., 2015; Weinl & Kudla, 2009). In the current study, we assessed unigenes related to calcium signaling in *G. littoralis*. Some of these genes, such as CBLs and CIPKs, which are associated with plant salt-tolerance, were significantly upregulated. These data provide valuable genes targets for further research on physiological function in response to salt stress in *G. littoralis*. We also found that phospholipase-related DEGs were upregulated in *G. littoralis* under NaCl treatment (Fig. 8, Table S9). Phospholipases hydrolyse phospholipids. Phospholipase Ds (PLDs) and their hydrolysis product phosphatidic acid (PA), act as essential signal transducers and regulators involved in hyperosmotic stress responses (Hong et al., 2016; Zhang et al., 2014b). Previous studies have reported that several PLDs were activated by salt stress (Wang et al., 2014). In *Arabidopsis*, a lack of *PLDa1* and *PLDa3* resulted in more sensitivity to salt stress and a lower PA accumulation, and the change of PA level was suggested to affect plant salt response ( Hong et al., 2008; Yu et al., 2010). PA acts as a second messenger by its direct interaction with functional proteins, such as mitogen-activated protein kinase 6 (MPK6) and microtubule-associated protein (MAP65-1), to modulate the catalytic activity of binding proteins in response to salt stress (Yu et al., 2010; Zhang et al., 2012b). Moreover, a phosphoinositide-specific phospholipase C (OsPLC1)-mediated Ca^2+^ signaling pathway is essential for controlling Na^+^ accumulation in the rice response to salt stress (Li et al., 2017b).

Plant transcription factors respond to various environmental stimuli such as salt, drought, cold, and hormones and regulate gene expression by binding to core regions of promoters (Yadav et al., 2011). Several types of transcription factors associated with salt response have been identified in plants, including the MYB, NAC, bZIP, AP2/ERF, WRKY, and bHLH families (Cui et al., 2013; Hartmann et al., 2015; He et al., 2005; Jiang, Yang & Deyholos, 2009; Tao et al., 2011; Yang, Dai & Zhang, 2012; Zhang et al., 2009). In this study, we identified 151 differentially expressed transcription factors in *G. littoralis* under NaCl treatment (Table S5), suggesting their potential function in the regulation of *G. littoralis* salt response. For example, MYB constituted a large proportion of the DEGs encoding transcription factors in this study (Fig. 7). Previous studies have reported that the overexpression of the *OsMYB3R-2*, *LcMYB1*, and *TaMYB56-B* genes confers enhanced salt tolerance in transgenic plants (Cheng et al., 2013; Dai et al., 2007; Zhang et al., 2012a), and *AtMYB20* and *AtMYB73* act as negative regulators to enhance salt resistance (Cui et al., 2013; Kim et al., 2013). The changes in expression of these transcription factors in *Glehnia littoralis* could indicate their functional mechanism in salt stress.

*G. littoralis* roots (Radix Glehniae) are rich in polysaccharides, phospholipids, coumarins, coumarin glycosides and polyacetylenes (Tomshich et al., 1997; Yuan et al., 2002). *G. littoralis* grows naturally in coastal regions, and is considered a high-quality medicinal herb in traditional Chinese medicine. Previous studies have shown that salt stress could increase various secondary metabolites in plants, such as flavonoids, jasmonic acid, polyphenol, and polyamine (Ali, 2003; Ksouri et al., 2007; Mutlu & Bozcuk, 2007; Pedranzani et al., 2003; Ramakrishna & Ravishankar, 2011). Flavonoids were increased in response to salt stress in *Hordeum vulgare* seedlings (Ali, 2003). Ksouri et al. (2007) reported that polyphenol content and antioxidant activities were related to salinity in halophyte *Cakile maritima*. Free and bound polyamine content have been shown to be induced under salt stress in *Helianthus annuus* roots (Mutlu & Bozcuk, 2007). In tomato, endogenous jasmonic acid was increased under salt stress (Pedranzani et al., 2003). Based on the habitat of *G. littoralis*, we hypothesize that its active compounds may be associated with environmental salt stress. In a previous study of *Peucedanum praeruptorum* (Umbelliferae), transcriptome sequencing and high-performance liquid chromatography coupled with electrospray-ionization quadrupole time-of-flight mass spectrometry (HPLC-Q-TOF-MS/MS)-based metabolomics datasets were constructed for investigating the genes involved in coumarin biosynthesis and transport, as well for compound identification (Zhao et al., 2015). In total, 40,952 unigenes and 19 coumarin compounds were obtained and a few unigenes were predicted to be related to the formation of the coumarin core compounds in *Peucedanum praeruptorum*. Therefore, the *G. littoralis* transcriptome sequencing dataset could be used in future research to provide essential genes related to metabolism, and investigate biosynthetic pathways of the active compounds of *G. littoralis*.

## Conclusions

In this study, we performed a comprehensive transcriptome analysis of *G. littoralis* in response to salt stress by Illumina 2000 sequencing. A significant number of unigenes, including DEGs, were identified and annotated. The DEGs dataset also provided candidate genes, which may be involved in salt tolerance or secondary metabolism pathways, to be used in subsequent functional analyses. These data will be helpful for future *G. littoralis* genomic studies, and will also be beneficial to studies on other species of Umbelliferae, and on halophytes.

##  Supplemental Information

10.7717/peerj.5681/supp-1 Table S1 qRT-PCR primers used in this study

10.7717/peerj.5681/supp-2 Table S2 Gene ontology (GO) enrichment analysis of unigenes

10.7717/peerj.5681/supp-3 Table S3 EuKaryotic Orthologous Groups (KOG) classification of assembled unigenes

10.7717/peerj.5681/supp-4 Table S4 Kyoto Encyclopaedia of Genes and Genomes (KEGG) metabolism pathway categories of total unigenes

10.7717/peerj.5681/supp-5 Table S5 Numbers of annotated genes and expressed features

10.7717/peerj.5681/supp-6 Table S6 GO enrichment analysis of differentially expressed genes (DEGs)

10.7717/peerj.5681/supp-7 Table S7 KEGG metabolism pathway categories of DEG unigenes

10.7717/peerj.5681/supp-8 Table S8 DEGs information in secondary metabolism pathways shown in heat map analysis

10.7717/peerj.5681/supp-9 Table S9 Signal transduction gene information shown in heat map analysis

## Additional Information and Declarations

### Data Availability

The following information was supplied regarding data availability: This transcriptome sequencing raw data were deposited into the National Center for Biotechnology Information (NCBI) Sequence Read Archive (SRA) (accession number: SRX547159). This Transcriptome Shotgun Assembly project has been deposited at DDBJ/EMBL/GenBank under the accession GGSB00000000. The version described in this paper is the first version, GGSB01000000.
